# The effect of bee drone brood on the motility and viability of stallion spermatozoa—an in vitro study

**DOI:** 10.1007/s11626-024-00918-y

**Published:** 2024-05-21

**Authors:** Michal Lenický, Ewelina Sidor, Lucia Dianová, Filip Tirpák, Nikola Štefunková, Małgorzata Dżugan, Marko Halo, Marko Halo, Tomáš Slanina, Iveta Urban, Denis Bažány, Agnieszka Greń, Shubhadeep Roychoudhury, Eric Rendon Schneir, Peter Massányi

**Affiliations:** 1 Institute of Applied Biology, Faculty of Biotechnology and Food Sciences, Slovak University of Agriculture, Tr. A. Hlinku 2, 94976 Nitra, Slovak Republic; 2 Department of Chemistry and Food Toxicology, Institute of Food Technology and Nutrition, University of Rzeszów, Ćwiklińskiej 1a St., 35-601, Rzeszów, Poland; 3 Doctoral School, University of Rzeszow, Rejtana 16C, 35-959, Rzeszow, Poland; 4 Division of Animal Sciences, University of Missouri, 920 East Campus Drive, Columbia, MO 65211-5300 USA; 5 Institute of Animal Husbandry, Faculty of Agrobiology and Food Resources, Slovak University of Agriculture, Tr. A. Hlinku 2, 94976 Nitra, Slovak Republic; 6 Institute of Biology, Pedagogical University of Krakow, Podchorazych 2, 30-084 Krakow, Poland; 7 Department of Life Science and Bioinformatics, Assam University, Silchar, India; 8 Faculty of Planning, The National University Agraria La Molina, Lima, Peru

**Keywords:** Spermatozoa, Motility, Viability, Stallion, Drone brood, Testosterone

## Abstract

Bee drone brood is a beehive by-product with high hormonal activity used in natural medicine to treat male infertility. The aim of the study was to assess the effect of drone brood on stallion spermatozoa during a short-term incubation for its potential use in the equine semen extenders. Three different forms of fixed drone brood (frozen (FR), freeze-dried (FD), and dried extract (DE)) were used. Solutions of drone brood were compared in terms of testosterone, protein, total phenolic content, and antioxidant activity. The stallion semen was diluted with prepared drone brood solutions. The computer-assisted semen analysis (CASA) method was employed to evaluate the movement characteristics of the diluted ejaculate. To determine spermatozoa viability, the mitochondrial toxicity test (MTT) and Alamar Blue test were performed. In terms of testosterone content and antioxidant activity, a close likeness between FR and FD was found whereas DE’s composition differed notably. FR had a positive effect mainly on progressive motility, but also on sperm distance and speed parameters after 2 and 3 h of incubation. On the contrary, FD and DE acted negatively, depending on increasing dose and time. For the first time, a positive dose-dependent effect of fixed drone brood on spermatozoa survival in vitro was demonstrated.

**Graphical Abstract:**

**Figure:**
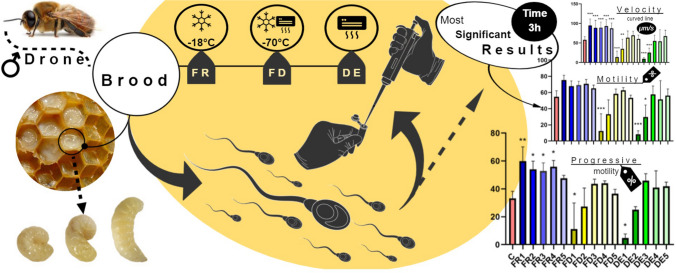

**Supplementary Information:**

The online version contains supplementary material available at 10.1007/s11626-024-00918-y.

## Introduction

Bee products have been used in natural medicine to support the treatment of various diseases for centuries. The most popular is honey, which, due to its biological properties, is often used to treat diseases of various origins (e.g., cold, sore throat, diabetes, hypertension). Research revealed that oral administration of honey enhances serum testosterone level in male rats and monkeys (Banihani 2019). The beehive, in addition to honey, offers other nutritive and therapeutic products, including the drone brood (male bee larvae). It is a product rich in proteins (40%), amino acids, hormones (testosterone, estradiol, progesterone), bioelements (selenium, potassium, phosphorus), and energy substrates (fructose, glucose, and sucrose) (Sawczuk *et al*. 2019; Sidor *et al*. 2021b). However, this bee product is unstable and requires immediate fixation after extraction from the comb. Various methods have been proposed for drone brood preservation which allows it to maintain its activity. Apart from hormonal activity, drone brood’s mineral composition suggests its supportive role in the management of male infertility. It may provide protection to the spermatozoa directly in the liquid seminal extender (Seres *et al*. 2014). The anabolic and androgenic hormone-like effects of bee drone brood have been mentioned in Eastern European and Asian folk medicines (Yemets 2020). So far, few studies have confirmed the biological effect of drone brood such as slowing down the course of peroxidation processes. However, its action on spermatozoa has not been described. A recent report elaborated on the effect of drone brood on the homeostasis and reproductive capacity of gilts (Loomis 2006).

In sustainable livestock production, one of the key strategies involves the use of the artificial insemination method. One of the basic goals of assisted reproduction is to maximize the viability of spermatozoa and minimize the damage caused by the handling or external environment so that the fertilizing ability of the spermatozoa is preserved to the highest extent (Bustani and Baiee 2021). Special media have been formulated to enhance the ability of spermatozoa to cope with issues like irregular pH, changes in osmolarity, lack of energy sources, oxidative stress, or membrane damage (Pezo *et al*. 2019). Liquid semen extenders purposed for short-term storage are well-established and widely used. Substances contained in equine extenders usually include the correct ratio of electrolytes and non-electrolytes that maintain osmotic balance, sugars to increase sperm motility, milk and egg yolk to protect against temperature changes, and other supplements. The extender based on skimmed milk is the most commonly used in chilled semen storage (Rečková *et al*. 2022). Attention is also paid to the search for new bioactive substances that effectively attenuate the aforementioned adverse factors, mainly substances rich in vitamins, polyphenols, carotenoids, flavonoids, isoflavonoids, and anthocyanins (Seddiki *et al*. 2017; Sidor *et al*. 2021a). The unique composition of drone brood has the potential to provide spermatozoa with nutrients and antioxidant agents. However, knowledge about the direct influence of drone brood on spermatozoa in vitro is not known yet and the information sources are limited. Demonstrating its protective effect on spermatozoa would allow the use of drone brood as a component of semen extenders, which may effectively prolong the viability of spermatozoa and thus improve the resulting chances of successful fertilization. This hypothesis inspired us to perform a pilot study in vitro and to treat spermatozoa of breeding stallions with drone brood. Additionally, the present study used various forms of drone brood: frozen, freeze-dried, and dried extract, to experimentally verify the potential of drone brood on spermatozoa.

## Material and methods

### Drone brood collection and processing

Drone brood samples were collected from three apiaries situated in the south-eastern part of Poland in June 2022 from the families of the *Apis mellifera carnica* breed. About 50 g of 7-d-old drone brood larvae was manually extracted from the working frame, immediately sealed in a sterile container, and transported to the laboratory at room temperature. After the pooling of three samples, the raw material (without any buffer) was homogenized using a tissue homogenizer (TH 02, Omni International, Kennesaw, GA). The homogenized sample was divided into three parts (50 g each): the first part was frozen at − 18°C (FR), the second at − 70°C, and then freeze-dried (FD), and the third aliquot was processed to dried extract (DE). Freeze-drying was carried out using the device Alpha 1–2, LD plus (Martin Christ Gefriertrocknungsanlagen GmbH, Osterode, Germany). Dehydration was carried out for 72 h by cooling the sample to − 55°C at a standard pressure of 0.1 bar. A dried extract (DE) of the drone brood was obtained from the FD drone brood. Briefly, 1 g of FD sample was mixed with 10 mL of distilled water, homogenized on a vortex, and left for 24 h at 4°C. After centrifugation (6000 rpm, 10 min; FC 5306, Ohaus, Naenikron, Switzerland), the supernatant was decanted and subjected to freeze-drying by the method described above whereas the sediment was refused. Both FD and DE samples were stored in a desiccator until the analysis. Before in vitro experiments, all drone brood samples were ground in a mortar. To create stock solutions, samples (FR, FD, DE) were suspended in 0.9% NaCl with the starting concentration (FR 8 mg/mL, FD 2.4 mg/mL, DE 7.2 mg/mL of 0.9% NaCl), which were calculated for the same weight of raw material taking into account the water loss during freeze-drying for FR and FD forms (60% and 5%, respectively) and the loss of solids refused as sediment during processing of DE.

### Drone brood analysis

The antioxidant activity of the stock solution of the three drone brood samples (FR, FD, and DE) was compared using standard 2,2-diphenyl-1-picrylhydrazyl (DPPH) and ferric reducing antioxidant power (FRAP) assays. The inhibition of DPPH radicals was measured (Sidor *et al*. 2021b). As a positive control, Trolox methanolic solution was applied. The results were expressed as nmol TE/mL of stock solution. The FRAP test was performed (Sidor *et al*. 2021b). Results were expressed as mmol Trolox equivalents (TE) per 1 mL of each stock solution (µmol/mL) from the calibration curve prepared for Trolox in the range 5–60 nmol/mL (*y* = 0.152*x*, *R*^2^ = 0.9989) (Budnikova and Mitrofanov 2021).

The total content of phenolic compounds was determined using the Folin–Ciocalteu reagent (Sidor *et al*. 2021b). The results were expressed as µg of gallic acid equivalents (GAE) per 1 mL of each stock solution (µg GAE/mL). The results were calculated based on a calibration curve prepared for gallic acid in the range of 0–125 µg/mL (*y* = 0.336*x*, *R*^2^ = 0.9914) (Sidor *et al*. 2021a).

Soluble protein fraction was determined by the Bradford method in prepared extracts. The results were calculated based on a calibration curve of 0–100 µg/per sample (*y* = 0.0551*x*, *R*^2^ = 0.9991). Bovine albumin was used as a standard protein (Sidor *et al*. 2021a).

The concentration of testosterone was demonstrated using immunoenzymatic ELISA test kits (abx574314) according to the manufacturer’s instructions (Abbexa, Cambridge, UK) as previously described (Sidor *et al*. 2021a). The results were expressed as pmol/mL (1 mL of appropriate stock solution).

All analyses of drone brood were performed in triplicates, and thus, the final value for each analysis of all three drone brood samples is expressed as mean ± SD.

### Semen collection and processing

The ejaculates were collected from clinically healthy 3- to 18-yr-old breeding stallions of Oldenburger and Holstein breeds (*n* = 4) bred in the district of Nitra, Slovakia. Stallions were handled carefully in accordance with the ethical guidelines of the Animal Protection Regulation of the Slovak Republic RD 377/12, complying with the European Union Regulation 2010/63. The experimental protocol was approved by the ethics committee of the Slovak University of Agriculture in Nitra, Slovakia. The stallions were at the time of the study in the breeding season and had their ejaculate collected at the same frequency, three times a week with 48 h of sexual abstinence between each collection. Collections for the purposes of this experiment were realized within 2 and half weeks. Stallions were housed in the same conditions and received identical feed. To collect the ejaculate, an artificial vagina (preheated and lubricated) was used (Colorado type, Minitube, Tiefenbach, Germany). Immediately after the collection of the ejaculate, sperm motility was measured directly at the collection site using the HUVESearch Stallion spermatozoa analyzer iSperm (HUVESearch, Pelt, Belgium). For this experiment, only ejaculates of the required quality (minimum 50% motility and a concentration of at least 100 × 10^6^ spermatozoa/mL) were used. The final number of semen samples used in the experiment was four (*n* = 4), each ejaculate from the different stallions. Subsequently, the ejaculate was transported to the laboratory in a thermally insulated container within 5 min. Immediately after collection, the ejaculates were diluted in a ratio of 1:3 in previously prepared and preheated (37°C) drone brood solutions (Table 1). Even though the concentrations of spermatozoa in ejaculates vary among stallions, the concentrations of collected ejaculates based on the results of the CASA (AndroVision software, Minitube, Tiefenbach, Germany) were very similar (Supplementary Table 1). This allowed to apply uniform dilution at a ratio of 1:3 (spermatozoa:drone brood solutions/saline) on all the semen samples subjected to the study to achieve a final concentration of approximately 100 × 10^6^. To express used ratio in exact volumes, 433 µL of semen sample was mixed with 867 µL of each drone brood solution described in Table 1. The final volume of all experimental samples was 1300 µL. The control sample consisted of 867 µL of 0.9% NaCl (Braun, B. Braun SE, Melsungen, Hessen, Germany) and 433 µL of semen sample. All samples were subjected to spermatozoa motility analysis at 0, 1, 2, 3, and 4 h of incubation at 37°C. To determine spermatozoa viability, the mitochondrial toxicity test (MTT) and Alamar Blue MT test were performed after 1 and 4 h of incubation.

**Table 1.** Concentrations of drone brood solution used for the study

Drone brood solution	Frozen (FR) [mg FR/mL 0.9% NaCl]	Freeze-dried [FD] [mg FD/mL 0.9% NaCl]	Dried extract [DE] [mg DE/mL 0.9% NaCl]
1	4.0	1.2	3.6
2	2.0	0.6	1.8
3	1.0	0.3	0.9
4	0.5	0.15	0.45
5	0.25	0.075	0.225

### Spermatozoa motility

The basic parameters of spermatozoa motility were determined using the computer-assisted semen analysis (CASA). The system uses an optical microscope, Olympus BX 51 (Olympus, Tokyo, Japan), in combination with AndroVision software (Minitube, Tiefenbach, Germany). Volume 10 μL of each sample was placed in a Makler counting chamber (10 μL, Sefi-Medical Instruments, Haifa, Israel) preheated to 37.5°C. Evaluation of spermatozoa was performed in five time periods (0, 1, 2, 3, 4 h). The optical system records 30 frames per second and the measurement was performed on at least three different fields within the counting chamber. Sperm quality was evaluated using the following parameters: total motility (MOT, %), progressive motility (PRO, %), velocity curved line (VCL, %), and distance curved line (DCL, %) (Halo *et al*. 2022; Massányi *et al*. 2008).

### Spermatozoa viability

A mitochondrial toxicity test was used to evaluate the viability of stallion spermatozoa. It is a colorimetric method based on the conversion of 3-(4,5-dimethylthiazol-2-yl)-2,5-diphenyltetrazolium bromide (MTT; Sigma-Aldrich, St. Louis, MO) to purple-colored formazan particles. The conversion is mediated by the mitochondrial enzyme succinate dehydrogenase, which is produced by mitochondria with an intact mitochondrial membrane (Supino 1995). The MTT stock solution consisted of 50 mg of 3-(4,5-dimethylthiazol-2-yl)-2,5-diphenyltetrazolium bromide (Sigma-Aldrich) dissolved in 10 mL of physiological solution (Braun, B. Braun Melsungen AG, Germany). A total of 100 µL of each sample was pipetted into a 96-well plate in triplicate. 20 µL of MTT solution was further added to the samples, followed by incubation for 1 h at 37°C. During incubation, an enzymatic reaction takes place, and is stopped by adding 40 µL of isopropanol (CentralChem, Bratislava, Slovakia). Consecutively, the plate was placed on a shaker for 15 min. Mitochondrial activity was determined by an ELISA reader (Multiskan FC, Thermo Fisher Scientific, Vantaa, Finland) in two wavelengths, namely 570 nm and 620 nm. Data were expressed as a percentage—compared to the metabolic activity of spermatozoa of the control group (Halo Jr *et al*. 2021a; b).

### Spermatozoa metabolic activity

The sensitive redox indicator Alamar Blue™ (Thermo Fisher Scientific, Invitrogen, Vantaa, Finland) was also used to determine effective concentrations of drone brood extract on stallion spermatozoa viability. The blue-colored resazurin was reduced to the form of resorufin, which is pink in color (Hamid *et al*. 2004; Rampersad 2012). Alamar Blue™ reagent solution consists of 400 µL of resazurin (AB, Thermo Fisher Scientific, Invitrogen, Vantaa, Finland) diluted in 9.6 mL of phosphate buffer (PBS, Sigma-Aldrich). Incubation was performed in a 96-well plate containing 100 µL of each sample. A total of 20 µL of Alamar Blue solution was added to the samples, followed by 1 h of incubation at 37°C. Subsequently, the entire plate was transferred to a shaker for 15 min. The metabolic activity ratio of cells was quantified using an ELISA reader (Multiskan FC, Thermo Fisher Scientific, Vantaa, Finland) at 570 nm and 620 nm. All analyses were performed in triplicate and the averaged results were expressed as a percent of the control group which was set to 100% (Jambor *et al*. 2022).

### Statistical analysis

Results demonstrating the biological activity of drone brood were obtained using one-way ANOVA followed by Tukey’s test, which compares the means of multiple groups (FD, FR, DE), and the results are expressed as the level of significant difference (*p* < 0.05). Calculations were performed using the Statistica 13.3 software (StatSoft, Tulsa, OK). Results of control and experimental groups from each of four analyzed semen samples were grouped according to the treatment with drone brood and analyzed as follows. A comparison between the control group and experimental groups was statistically estimated using the GraphPad Prism program (GraphPad Software, La Jolla, CA). Descriptive statistical parameters (mean, standard deviation (SD)) were determined using the one-way ANOVA. The statistical difference was expressed as follows: ***p < 0.001, **p < 0.01, and *p < 0.05. Results were interpreted as means and expressed with standard deviation (SD).

## Results

### Biological activity of drone brood

Analysis of antioxidant activity by DPPH and FRAP methods revealed similar (*p* > 0.05) biological potential of the two different drone brood stock solutions FR and FD. Antioxidant activity determined by FRAP assay was higher (*p* < 0.05) for DE, as compared to that for FR and FD groups. Interestingly, the FRAP reducing potential of DE was 39.2% higher than that of FR, which was characterized by a much higher content of polyphenolic compounds (by 132.5% compared to FR) (Table 2). Similarly, the content of total protein was similar in FR and FD groups; however, it was significantly higher (*p* < 0.05) in the DE group. On the other hand, soluble protein fraction in drone brood extracts was higher in FD and DE groups as compared with that in the FR group (*p* < 0.05). Testosterone levels were similar in FR and FD groups, but DE significantly (*p* < 0.05) differed.

**Table 2.** Antioxidant activity, protein content, and testosterone level of various forms of drone brood stock solutions

	Frozen (FR) [mg FR/mL 0.9% NaCl]	Freeze-dried [FD] [mg FD/mL 0.9% NaCl]	Dried extract [DE] [mg DE/mL 0.9% NaCl]
DPPH [nmolTE/mL]	6.91 ± 0.97a	7.74 ± 1.14a	7.77 ± 1.34a
FRAP [μmolTE/mL]	0.125 ± 0.014a	0.106 ± 0.040a	0.174 ± 0.012b
TPC [μg GAE/mL]	19.61 ± 1.21a	17.30 ± 1.84a	45.60 ± 8.16b
Soluble protein fraction [%]	2.04 ± 0.15a	3.45 ± 0.19b	3.40 ± 0.11b
Testosterone [pmol/mL]	0.092 ± 0.001a	0.086 ± 0.002a	0.131 ± 0.001b

^a,b^Means marked with different *letters* in *rows* are statistically significant (*p* < 0.05, Tukey’s test)

*DPPH* 2,2-diphenyl-1-picrylhydrazyl, *FRAP* ferric reducing antioxidant power, *TPC* total phenolic content

### Spermatozoa motility parameters-Total motility

**Figure 1.**
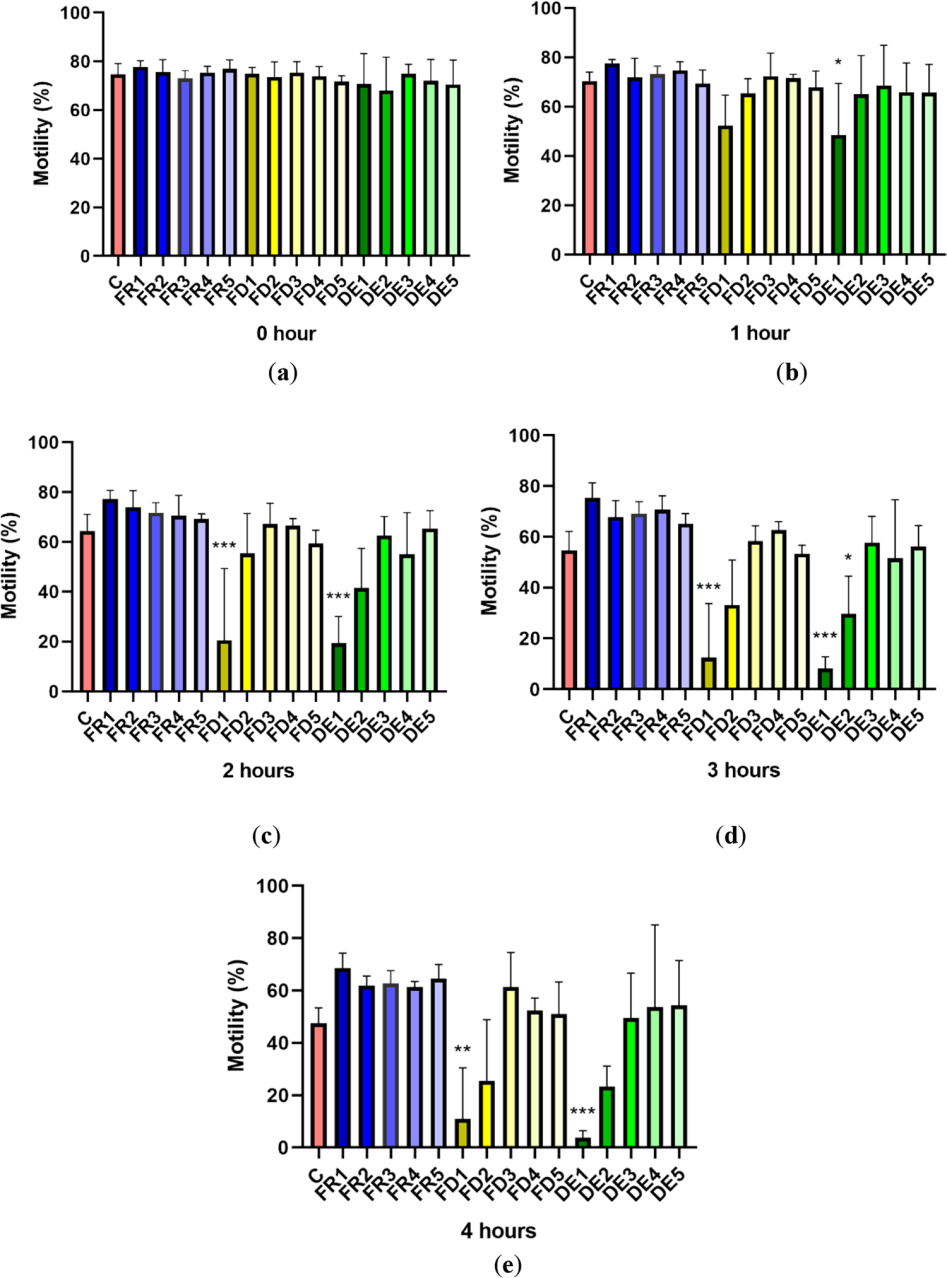
Effect of drone brood solution on total spermatozoa motility (%) in stallion after 0 (***a***), 1 (***b***), 2 (***c***), 3 (***d***), and 4 (***e***) hours of treatment. C, control; FR, frozen; FD, freeze-dried (lyophilizate); DE, dry extract. FR1 4.0 mg/mL, FR2 2.0 mg/mL, FR3 1.0 mg/mL, FR4 0.5 mg/mL, FR5 0.25 mg/mL, FD1 1.2 mg/mL, FD2 0.6 mg/mL, FD3 0.3 mg/mL, FD4 0.15 mg/mL, FD5 0.075 mg/mL, DE1 3.6 mg/mL, DE2 1.8 mg/mL, DE3 0.9 mg/mL, DE4 0.45 mg/mL, DE5 0.225 mg/mL of drone brood solutions. The levels of significance were set at ***p < 0.001, **p < 0.01, and *p < 0.05.

In the initial interval, immediately after the dilution of ejaculate with drone brood extract (time 0), total motility did not differ in the tested groups compared to the control (*p* > 0.05). However, after an hour of incubation at 37°C (time 1), a significant decrease in total motility (*p* < 0.05) was observed in the DE1 group (48.492 ± 20.925%). Other tested groups did not show any significant difference after 1 h compared to the control. After 2 h (time 2), there was a decrease (*p* < 0.001) in total motility of spermatozoa in the groups DE1 (19.287 ± 10.726%) and FD1 (20.4850 ± 28.821%) as compared to the control, while in the other groups, motility differed just slightly with no statistical significance. A similar tendency was noted after 3 h (time 3) when a significant decrease (*p* < 0.001) of total motility was recorded in the groups FD1 (12.425 ± 21.283%) and DE1 (8.327 ± 4.428%) (*p* < 0.001) together with DE2 (29.610 ± 14.489%) (*p* < 0.05), in comparison to the control. Four hours of in vitro incubation of stallion spermatozoa treated with drone brood extract showed a significant decrease in FD1 (10.927 ± 19.491%; *p* < 0.01) and DE1 (3.727 ± 2.736%; *p* < 0.001) groups as compared to the control. Incubation intervals of 3 and 4 h suggested a mild beneficial effect of FR on sperm motility, but no significance was detected (Fig. 1).

### Progressive motility

**Figure 2.**
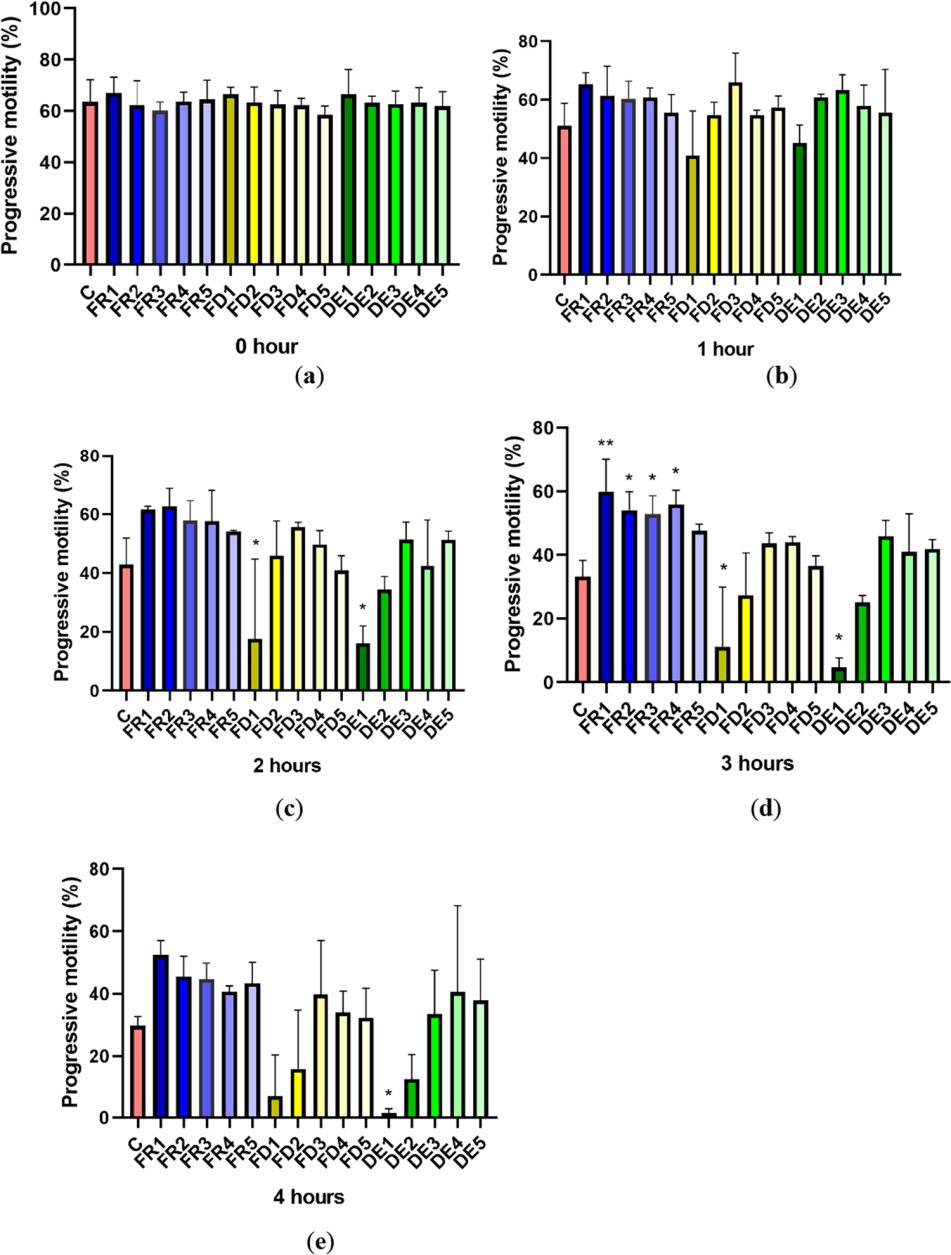
The effect of drone brood solution on the progressive (PRO) spermatozoa motility (%) in 0 (***a***), 1 (***b***), 2 (***c***), 3 (***d***), and 4 (***e***) hours. C, control; FR, frozen; FD, freeze-dried (lyophilizate); DE, dry extract. FR1 4.0 mg/mL, FR2 2.0 mg/mL, FR3 1.0 mg/mL, FR4 0.5 mg/mL, FR5 0.25 mg/mL, FD1 1.2 mg/mL, FD2 0.6 mg/mL, FD3 0.3 mg/mL, FD4 0.15 mg/mL, FD5 0.075 mg/mL, DE1 3.6 mg/mL, DE2 1.8 mg/mL, DE3 0.9 mg/mL, DE4 0.45 mg/mL, DE5 0.225 mg/mL of drone brood solutions. The level of significance was set at ***p < 0.001, **p < 0.01, and *p < 0.05.

Progressive motility showed no statistical difference (*p* > 0.05) at time 0 and time 1 in any of the experimental groups in comparison to the control. Nevertheless, after 2 h of in vitro treatment with drone brood extract (time 2), the same tendency as in the case of total motility was detected, with a significant decrease (*p* < 0.05) in DE1 (16.076 ± 5.862%) and FD1 (17.576 ± 27.093%) groups, as compared to the control (42.8267 ± 9.088%). Contrary, after 3 h of in vitro treatment (time 3), a stimulatory effect of the frozen extract of drone brood (FR1 to FR4) on stallion spermatozoa was observed, as compared to the control (Fig. 2). Furthermore, the highest concentrations of lyophilized and dried drone brood extracts—FD1 (11.240 ± 18.673%) and DE1 (4.766 ± 2.911%)—showed significant declines in progressive motility when compared to the control (33.170 ± 5.139) (*p* < 0.05). Likewise, a significant decrease (*p* < 0.05) of progressively motile spermatozoa was monitored in the group DE1 (1.407 ± 1.4527%) after 4 h of incubation (time 4) comparing with the control (29.777 ± 2.944%). Other experimental groups did not express any statistically significant variations at time 4.

### Distance curved line (DCL)

**Figure 3.**
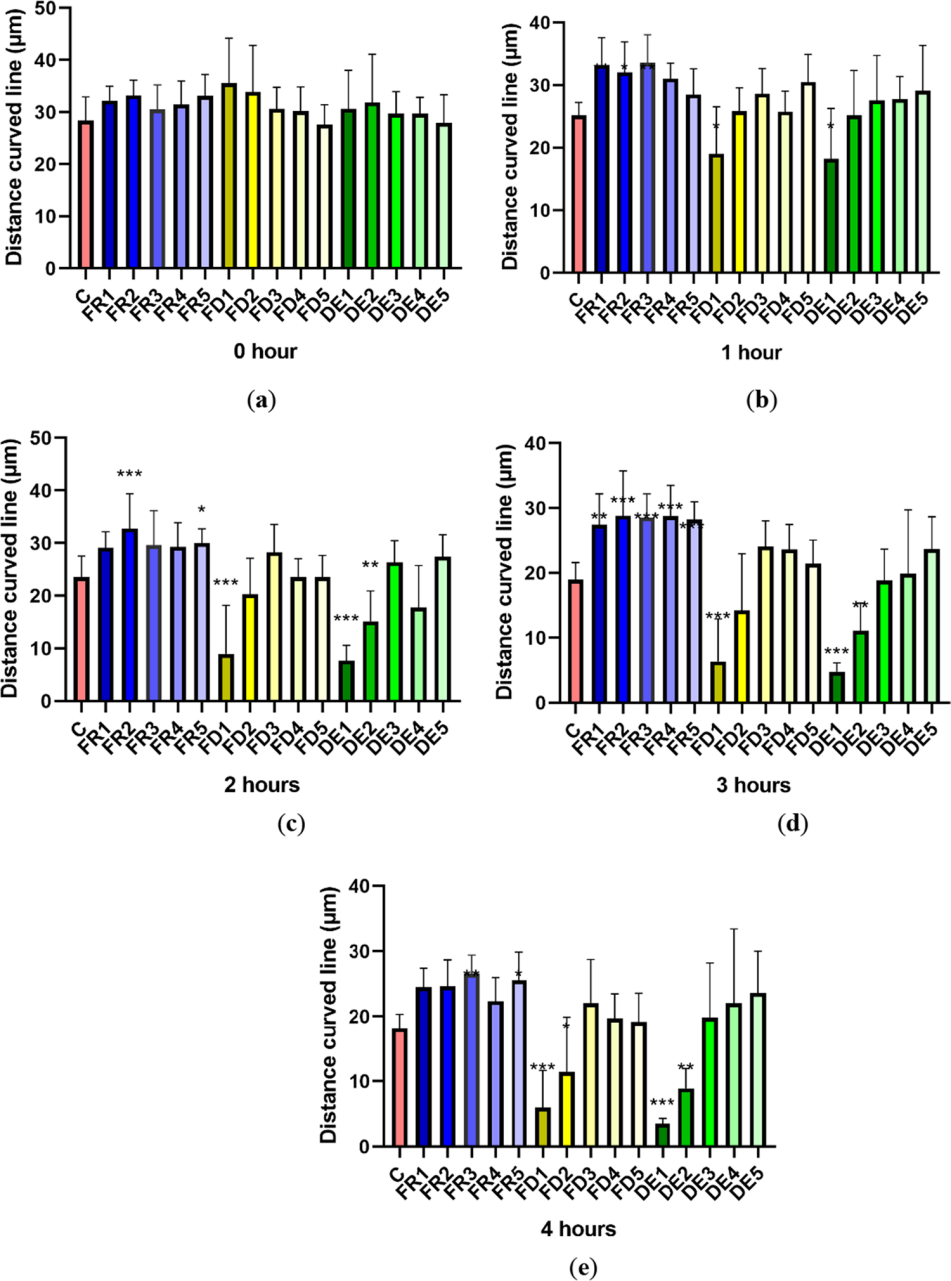
The effect of drone brood solution on the spermatozoa distance curved line (DCL) (µm) in 0 (***a***), 1 (***b***), 2 (***c***), 3 (***d***), and 4 (***e***) hours. C, control; FR, frozen; FD, freeze-dried (lyophilizate); DE, dry extract. FR1 4.0 mg/mL, FR2 2.0 mg/mL, FR3 1.0 mg/mL, FR4 0.5 mg/mL, FR5 0.25 mg/mL, FD1 1.2 mg/mL, FD2 0.6 mg/mL, FD3 0.3 mg/mL, FD4 0.15 mg/mL, FD5 0.075 mg/mL, DE1 3.6 mg/mL, DE2 1.8 mg/mL, DE3 0.9 mg/mL, DE4 0.45 mg/mL, DE5 0.225 mg/mL of drone brood solutions. The level of significance was set at ***p < 0.001, **p < 0.01, and *p < 0.05.

In the initial measurement (time 0), we observed no significant differences (*p* > 0.05) in all groups compared to the control. However, after the first hour of incubation at 37°C (Fig. 3), a significant increase (*p* < 0.01) in the distance in FR1 (33.221 ± 4.354 μm), FR3 (33.597 ± 4.438 μm), and in the group FR2 (32.048 ± 4.869 μm; *p* < 0.05) was observed. Groups FD1 (19.000 ± 7.534 μm) and DE1 (18.2042 ± 8.092 μm) showed a significant decrease (*p* < 0.05) in the curved line distance. Experimental groups FD1 (8.997 ± 9.181 μm) and DE1 (7.699 ± 2.905 μm) displayed a significant decrease (*p* < 0.001) in sperm distance swam after 2 h. The same incubation time negatively affected DCL in DE2 (15.135 ± 5.785 μm) (*p* < 0.01). In contrast, in the groups FR2 (32.782 ± 6.607 μm) and FR5 (30.039 ± 2.678 μm), a significant increase was observed (*p* < 0.001 and *p* < 0.05, respectively). Frozen drone brood extracts exhibited their full potential after 3 h of in vitro incubation when a significant increase (*p* < 0.001) in the distance of the curved line of stallion spermatozoa was observed in FR2 (28.854 ± 6.889 μm), FR3 (28.597 ± 3.616 μm), FR4 (28.772 ± 4.479 μm), FR5 (28.204 ± 2.793 μm), and in FR1 (27.418 ± 4.805 μm; *p* < 0.01). A significantly negative effect was shown in groups FD1 (6.391 ± 6.511 μm; *p* < 0.001), DE1 (4.784 ± 1.358 μm; *p* < 0.001), and DE2 (11.080 ± 4.312 μm; *p* < 0.01). With increasing time (time 4 h), the inhibitory effect of lyophilized (FD) and dried extracts (DE) was confirmed. A negative significant (*p* < 0.001) alteration was registered in FD1 (5.993 ± 5.650 μm) and DE1 (3.477 ± 0.852 μm) and also in samples treated with lower drone brood concentrations: FD2 (11.451 ± 8.454 μm;* p* < 0.05) and DE2 (8.915 ± 3.034 μm; *p* < 0.01). Groups FD3, FD4, and FD5; and DE3, DE4, and DE5 did not show statistically significant differences, although samples with lower concentrations of dried and lyophilized extracts suggest more promising values: FD3 (22.024 ± 6.731 μm), DE4 (22.065 ± 11.340 μm), and DE5 (23.661 ± 6.34 μm) (Fig. 3e). Notably, the frozen extract (FR) maintains strikingly good values in all concentrations (FR3 (26.594 ± 2.814 μm) and FR5 (25.586 ± 4.304 μm)) even after 4 h of cultivation. The significant increase (*p* < 0.01 and *p* < 0.05, respectively) in the groups FR3 (26.594 ± 2.814 μm) and FR5 (25.586 ± 4.304 μm) was monitored.

### Velocity curved line (VCL)

**Figure 4.**
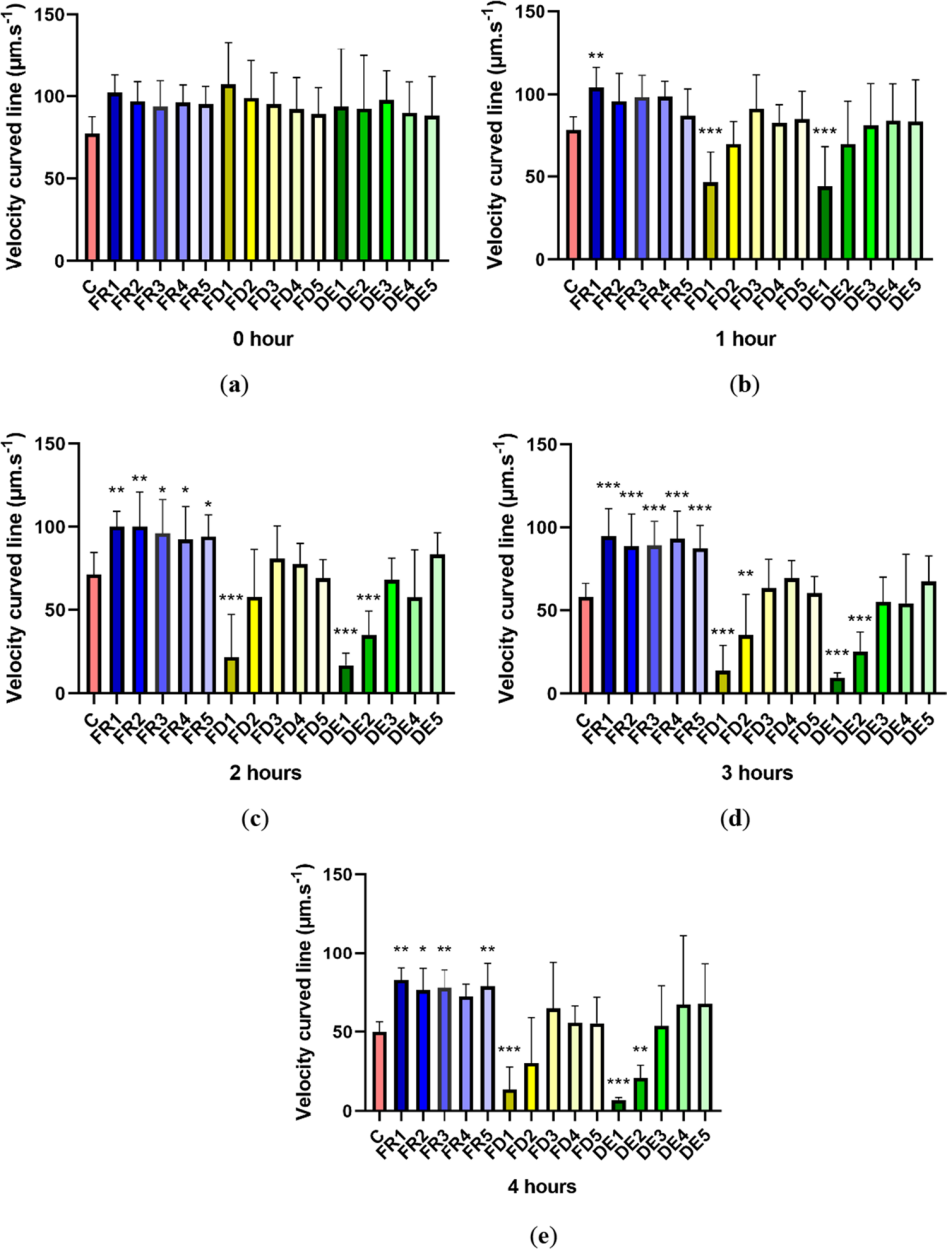
The effect of drone brood solution on the spermatozoa velocity curved line (VCL) spermatozoa motility (µm/s) in 0 (***a***), 1 (***b***), 2 (***c***), 3 (***d***), and 4 (***e***) hours. C, control; FR, frozen; FD, freeze-dried (lyophilizate); DE, dry extract. FR1 4.0 mg/mL, FR2 2.0 mg/mL, FR3 1.0 mg/mL, FR4 0.5 mg/mL, FR5 0.25 mg/mL, FD1 1.2 mg/mL, FD2 0.6 mg/mL, FD3 0.3 mg/mL, FD4 0.15 mg/mL, FD5 0.075 mg/mL, DE1 3.6 mg/mL, DE2 1.8 mg/mL, DE3 0.9 mg/mL, DE4 0.45 mg/mL, DE5 0.225 mg/mL of drone brood solutions. The level of significance was set at ***p < 0.001, **p < 0.01, and *p < 0.05.

Velocity curved line (VCL) results are in many ways reflective of DCL results. At time 0, no significant differences (*p* > 0.05) were observed. Following the first hour, a significant (*p* < 0.01) increase in sperm speed in the FR1 group (104.205 ± 12.147 μm/s) was detected (Fig. 4, time 1). Groups of the highest concentrations of lyophilized, FD1 (46.662 ± 18.661 μm/s) and dried extract DE1 (44.142 ± 24.370 μm/s), showed a significant decrease (*p* < 0.001) of VCL. Gradually with increasing incubation time (time 2 and time 3), we observed the stimulating effects of frozen extracts, while the spermatozoa diluted in groups FR1–5 reached a better velocity compared to the control and experimental samples enriched with lyophilized and dried drone brood extract. After 2 h of incubation (Fig. 4, time 2), we observed a significant increase (*p* < 0.01) in the velocity compared to the control in groups FR1 (100.174 ± 9.196 μm/s) and FR2 (100.132 ± 20.766 μm/s) as well as (*p* < 0.05) in groups FR3 (96.183 ± 20.220 μm/s), FR4 (92.449 ± 19.749 μm/s), and FR5 (94.040 ± 13.128 μm/s). On the contrary, FD1, DE1, and DE2 showed significantly lower values (*p* < 0.001) than the control after 2 h of incubation. The data after 3 h display a similar trend as at 2 h, however with major differences between individual experimental groups and the control. In all concentrations of the frozen extract–treated semen (FR1, FR2, FR3, FR4, FR5), stallion spermatozoa achieve a significantly higher (*p* < 0.001) velocity compared to the control (Fig. 4, time 3). Significantly lower values (*p* < 0.001 and *p* < 0.01) were recorded for FD1 and FD2 as well as DE1 and DE2. This indicates that even lower concentrations of the lyophilized and dried extracts have a cytotoxic character with increasing time. Following 4 h of incubation, the differences between the individual groups diminished, but compared to the control, experimental groups had a significantly higher VCL in groups FR1, FR3, and FR5 (*p* < 0.01) as well as in FR2 (*p* < 0.05). At the same time, we observed a significantly reduced VCL in groups FD1 (*p* < 0.001), DE1 (*p* < 0.001), and DE2 (*p* < 0.01).

### Spermatozoa viability

**Figure 5.**
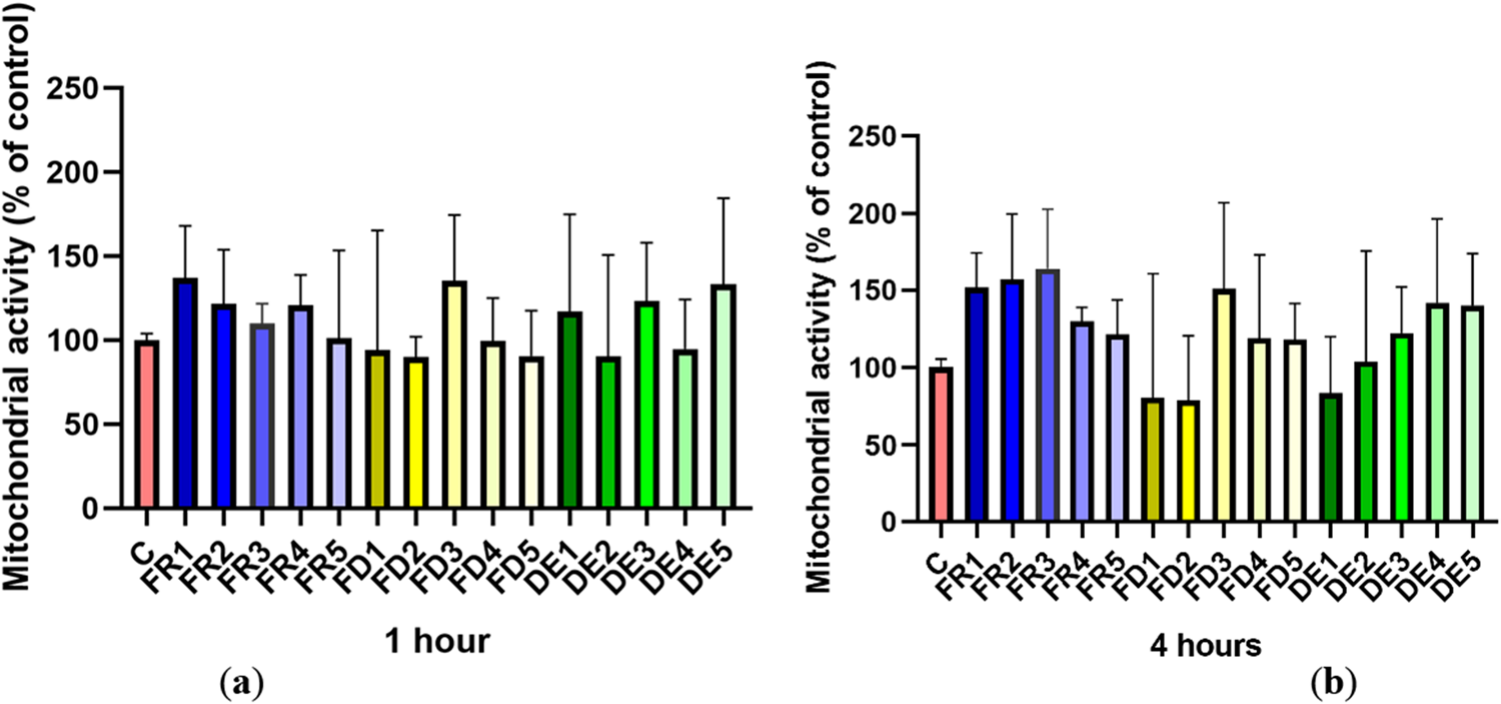
The effect of drone brood solution on the mitochondrial activity of spermatozoa in 1 (***a***) and 4 (***b***) hours. C, control; FR, frozen; FD, freeze-dried (lyophilizate); DE, dry extract. FR1 4.0 mg/mL, FR2 2.0 mg/mL, FR3 1.0 mg/mL, FR4 0.5 mg/mL, FR5 0.25 mg/mL, FD1 1.2 mg/mL, FD2 0.6 mg/mL, FD3 0.3 mg/mL, FD4 0.15 mg/mL, FD5 0.075 mg/mL, DE1 3.6 mg/mL, DE2 1.8 mg/mL, DE3 0.9 mg/mL, DE4 0.45 mg/mL, DE5 0.225 mg/mL of drone brood solutions. The level of significance was set at ***p < 0.001, **p < 0.01, and *p < 0.05.

MTT assay did not reveal any statistically significant difference (*p* > 0.05) in the viability of the spermatozoa at observed time intervals of 1 (time 1) and 4 (time 4) hours of drone brood treatments—FR, FD, and DE (Fig. 5).

### Spermatozoa metabolic activity

**Figure 6.**
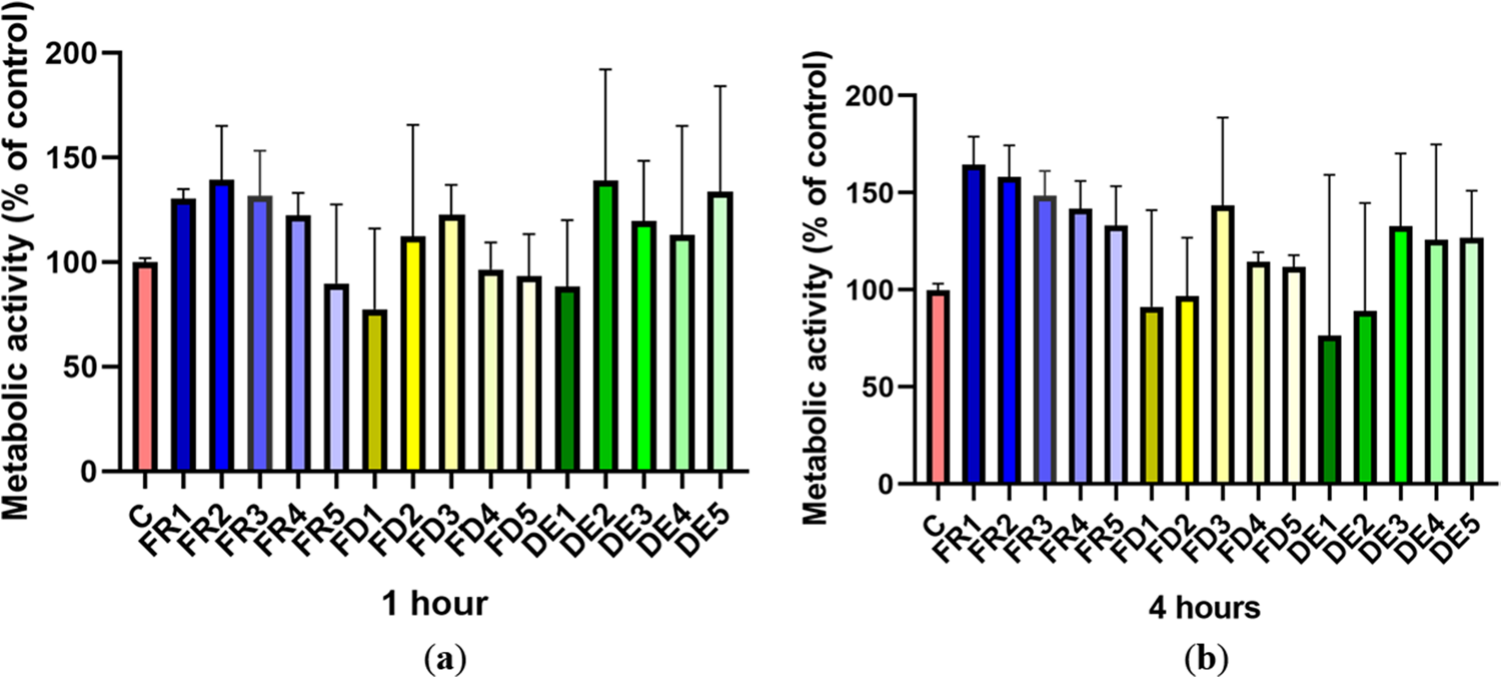
The effect of drone brood solution on the metabolic activity of spermatozoa in 1 (***a***) and 4 (***b***) hours. C, control; FR, frozen; FD, freeze-dried (lyophilizate); DE, dry extract. FR1 4.0 mg/mL, FR2 2.0 mg/mL, FR3 1.0 mg/mL, FR4 0.5 mg/mL, FR5 0.25 mg/mL, FD1 1.2 mg/mL, FD2 0.6 mg/mL, FD3 0.3 mg/mL, FD4 0.15 mg/mL, FD5 0.075 mg/mL, DE1 3.6 mg/mL, DE2 1.8 mg/mL, DE3 0.9 mg/mL, DE4 0.45 mg/mL, DE5 0.225 mg/mL of drone brood solutions. The level of significance was set at ***p < 0.001, **p < 0.01, and *p < 0.05.

Reflecting the results of MTT assay, the metabolic activity of spermatozoa assessed by the Alamar Blue test did not reveal any statistically significant difference (*p* > 0.05) at observed time intervals of 1 (time 1) and 4 (time 4) hours of drone brood treatments—FR, FD, and DE (Fig. 6). However, on a closer look, a similar declining trend, mimicking total and progressive motility, was observed in groups FD1 and DE1, although the differences were not statistically significant.

## Discussion

Usage of drone brood opens a range of new possibilities as a unique source of proteins and a high content of essential amino acids, specific enzymes, and sterols (Budnikova and Mitrofanov 2021). Beekeepers systematically remove drone cells from the hives for several reasons. One of the understandable reasons is safety of the entire hive. Varroa parasite prefers to invade the cells of the brood especially because the brood cells have a longer time of development than the cells of the workers and this way can cause more damage to the hive. Moreover, drone brood cells are several times larger than the worker cells, and they also consume a considerable amount of honey from the hive, thereby diminishing honey production (Ruffinengo *et al*. 2014; Rutka *et al*. 2021). Drone broods must be removed from the hives for the above-mentioned reasons, but at the same time, based on its rich composition of various nutrients, drone brood is a highly desired by-product of beekeeping. The aim of this study was to verify the potential of drone broods, processed by three different methods, as an additive to semen extenders to supplement and nourish equine spermatozoa. Stallion spermatozoa are very vulnerable, even in the first hours after the collection. This means that during the transport of semen to mare, the viability of spermatozoa over time decreases and the resulting ability to fertilize is reduced; therefore, the use of nourishing supplement in the semen extender is highly recommended (Tirpák *et al*. 2021a).

It is important to note that a drone brood is also characterized by antioxidant activity, although depending on several factors as, for example, the method of extraction or the method of preservation. After lyophilization, the antioxidant activity of drone brood decreases by up to 50% compared to the fresh homogenate (Budnikova and Mitrofanov 2021). Contrariwise, upon freezing, the decrease has only been minimal (by 1.8%) as compared to the raw homogenate. During lyophilization, drone brood acquires a porous structure with high absorption capacity, which leads to the binding of oxygen and moisture from the environment and induces oxidative reactions that significantly degrade the bioactive components of drone brood. Budnikova and Mitrofanov (2021) therefore postulated that the best way to stabilize the homogenate of drone brood is freezing, as it ensures sufficient fixation of its bioactive substances. According to Kaneko and Serikawa (2012), lyophilization is considered the mildest and least effective method of preserving not only food and vaccines but also mammalian spermatozoa. However, effectiveness of the lyophilization procedure and the biological value of the cells subjected to this process are determined by number of parameters, including the selection of the appropriate medium, the lyoprotectants and cryoprotectants used, and the selection of appropriate conditions during the procedure itself (e.g., pressure, temperature, drying time), as well as the proper storage of the material after the lyophilization process (Merivaara *et al*. 2021). When we compared frozen and freeze-dried samples during chemical analysis, similarities were observed in the tested parameters (testosterone, polyphenol, and antioxidant activity). In this study, the antioxidant capacity was evaluated using the DPPH and FRAP methods, while other authors used HPLC. Despite the effectiveness of lyophilization, our results clearly indicate a higher efficiency of processing drone broods by the freezing method, similar to Budnikova and Mitrofanov (2021). We achieved a better protective and stimulative effect on the parameters of spermatozoa (progressive motility, DCL, and VCL) with the FR extract than with the FD extract (Budnikova and Mitrofanov 2021). This confirms that precise assessment of bioactivity using only physical and chemical parameters of drone brood can be inadequate and a study on living cells is required to validate the claimed beneficial effects. The results of this study indicate the highest antioxidant activity in frozen extracts, which we consider to be a possible reason for the highest effectiveness of this extract on the motility parameters of stallion spermatozoa.

High antioxidant activity is very important for stallion ejaculate as stallion spermatozoa are susceptible to oxidative stress when overproduction of reactive oxygen species (ROS) leads to redox dysregulation. This represents a major issue, as most aspects of the proper functioning of spermatozoa are regulated by the redox system, and thus, spermatozoa may easily become damaged by ROS (Peña *et al*. 2019; Tirpák *et al*. 2021a).

Observed differences in drone brood effect may be due to the better solubility of the DE form compared to the FR and FD that formed the suspensions. These differences in the biological activity of tested drone brood solutions observed in vitro indicate their diverse chemical compositions, especially in the case of DE fraction (Sidor *et al*. 2021b); thus, their effect on spermatozoa may slightly differ. Several studies have described the benefits of bee products, including honey, propolis, and royal jelly, as a natural cryoprotectant for maintaining semen quality like kinetic parameters, sperm cell membrane and DNA integrity, and sperm morphology when added to semen cryopreservation media and liquid storage media (Hashem *et al*. 2021). Honey has been studied as the medium in which spermatozoa are diluted for assisted reproductive techniques (ART), and was found to protect spermatozoa against physicochemical stresses and prevent alterations to their structure and function (El-Nattat *et al*. 2016; Chung *et al*. 2019). Other studies have reported the beneficial effects of including royal jelly in sperm cell processing media on spermatozoa quality and, subsequently, fertility in some mammalian species, including rams (Moradi *et al*. 2013), goats (Alcay *et al*. 2017), and buffalo bulls (Shahzad *et al*. 2016). The protective role of royal jelly was mainly ascribed to its unique amino acid profile (Kodai *et al*. 2007). However, high concentrations of royal jelly produced negative impacts on sperm cell quality (Moradi *et al*. 2013); hence, careful consideration of the final concentration of added substance to the media is essential. Cited reports are in line with our findings, which may be explained based on the similarity of royal jelly and drone brood composition to a large extent (Sidor *et al*. 2021b). In the present study, higher doses of concentrated FD and DE samples of drone brood extracts (1.2 mg/mL and 3.6 mg/mL, respectively) exerted a rather negative effect on the motility parameters of stallion spermatozoa.

## Conclusions

The use of artificial insemination in horses brings numerous breeding as well as economic benefits. For this reason, it is important to engage in research of new (particularly natural) substances that can enhance the effectiveness of semen extenders. Interest in the use of natural substances is favorable when the use of natural alternatives is cost-effective and brings along the benefit of potentially better biocompatibility. Moreover, the use of natural substances perfectly aligns with the practices used in sustainable agriculture. Based on the results of the present study, it may be concluded that the concentration and method of extraction of drone brood are crucial for its effect on the stallion spermatozoa. Drone brood prepared by the freezing method (FR) exerted a positive impact on stallion spermatozoa, especially on progressive motility as well as on distance and velocity parameters (DCL and VCL) after 2 and 3 h of treatment in vitro. On the other hand, drone brood prepared by lyophilization (FD) and drying (DE), especially in the highest administered doses, resulted in negative influence on the observed ejaculate characteristics with increasing time of treatment. The parameter of total motility indicated that drone brood extracts have an inhibitory rather than a stimulatory effect on the sperm quality. These results indicate that, in addition to the concentration itself, it is very important to pay attention to the method of extraction of drone brood. Further detailed and systematic research is needed to confirm the results of this study as well as to better understand the effect of the drone brood extracts on stallion spermatozoa and mammalian spermatozoa in general. The current in vitro study could lay the foundation for further research and development leading to the use of drone brood as an additive to stallion semen extenders to maintain the quality of insemination doses.

### Supplementary Information

Below is the link to the electronic supplementary material.

Supplementary file1 (DOCX 14 KB)

## Data Availability

Data used and analyzed during this study are available from the corresponding author upon reasonable request.
