# Supplementary material for: The effect of bee drone brood on the motility and viability of stallion spermatozoa—an in vitro study
Source: In Vitro Cell Dev Biol Anim. 2024 May 21;60(6):596–608. doi: 10.1007/s11626-024-00918-y (PMC11286683; doi:10.1007/s11626-024-00918-y)
Supplement: Supplementary file 1 — Supplementary file1 (DOCX 14 KB) [file 11626_2024_918_MOESM1_ESM.docx]

**Supplementary table 1.:** Evaluation of the stallion semen prior to use in the study

| **Sample** | **Volume**  **[mL]** | **Concentration**  **[10^6^/mL]** | **Motility**  **[%]** |
| --- | --- | --- | --- |
| 1 | 25 | 289 | 65 |
| 2 | 25 | 280 | 73 |
| 3 | 25 | 267 | 70 |
| 4 | 15 | 351 | 78 |

The semen was evaluated right after the collection to ensure the specimen met the minimum criteria (sperm concentration>100x10^6^/mL and motility>50%) to be subjected to the study. This basic evaluation was performed using Stallion spermatozoa analyzer iSperm (HUVEsearch, Pelt, Belgium) which provides reliable results, however expressed only as round numbers.
